# Recent Research

**Published:** 1890-08-02

**Authors:** 


### RECENT RESEARCH.

The aphorism that little fleas have lesser fleas to bite them, and so *ad infinitum*, has been long accepted as a truism, but these little fleas are external to the body, and not in the interior. We have long known there are worms of different varieties which are sufficiently depraved to inhabit the vilest parts of our system. And we have perforce been obliged to accept the trichinæ as invaders of our muscular structure, and the *filariæ sanguinis hominis* as revellers in our blood. In short, if we gave a list of the numerous parasites which prey on the human frame, our readers might fairly exclaim, "Oh! what a tale of woe!" All this, however, is distanced when we find just published "A Report on the Conflict between the Organism and the Microbe." This report is by Mr. E. H. Hankin, B.A., of St. John's College, Cambridge, and we are sorry to say, it seems unquestionable that there is only too frequently going on in these vile bodies of ours a war to the very death between microbes which have gained entrance from without, and which have increased and multiplied with almost incredible rapidity, and the molecules, or living atoms of our body, on which these microbes or their products prey. But Mr. Hankin tells us it has been proved that "immunity against various diseases can be produced by the injection of the soluble products of the life of microbes." In other words, "chemical immunity" as distinguished from "vaccinal immunity," may be produced by the injection of attenuated virus. And it is somewhat reassuring to learn that there is a "bacteria-killing power" in the body. The occasional recovery of human beings from anthrax is noted as an instance of this bacteria-killing power, and further, the power which an animal possesses so long as it is alive of complete immunity against decay-producing bacteria. Mr. Hankin says that the only poisons known to have the power of aiding the existence of bacteria in the living body are not alkaloids but proteids. This fact gives probability to the idea that not ptomaines but poisonous proteids are the substances concerned in aiding the entry of disease-producing bacteria into the body and consequently, when tolerance is acquired of them, of giving immunity against disease. These considerations led Mr. Hankin to attempt to isolate the albumose from anthrax cultures. By means of the albumose he was able to produce immunity against anthrax in rabbits and in mice. Mr. Hankin's method of preparing the anthrax albumose is given in the *British Medical Journal* of July 12 last. And he also states that he has isolated from lymphatic glands a globulin, which has the power of killing anthrax bacilli. This globulin he says is identical with a proteid that Halliburton has isolated from lymph cells. Mr. Hankin further adds that the action of cell globulin is similar to the bacteria-killing activity that fresh blood serum has been shown to possess. The fact that he "has succeeded in obtaining immunity with the anthrax albumoses is a proof that these bodies form one of the factors in the conflict between the organisms and the microbe." Mr. Hankin's paper is very suggestive, and may lead to further discoveries; but neither rabbits nor mice are human beings, and the human body is not a test-tube.

